# Effects of Selenium Nanoparticles and Sodium Selenite Supplementation on Cryopreserved Ram Sperm Quality, Oxidative Status, and PRDX5 Gene Expression

**DOI:** 10.3390/ani16030457

**Published:** 2026-02-01

**Authors:** Cumali Kaya, Cansu Can, Burcu Esin, Emre Dünder, Mesut Çevik, Melih Akar

**Affiliations:** 1 Department of Animal Reproduction and Artificial Insemination, University of Ondokuz Mayis, 55200 Samsun, Türkiye; cumali.kaya@omu.edu.tr (C.K.);; 2 Department of Medical Biology, Faculty of Medicine, University of Ondokuz Mayis, 55200 Samsun, Türkiye; 3 Department of Statistics, Faculty of Science, University of Ondokuz Mayis, 55200 Samsun, Türkiye; 4 Department of Production Animal Medicine, Faculty of Veterinary Medicine, University of Helsinki, 00014 Helsinki, Finland

**Keywords:** cryopreservation, nanoparticle selenium, PRDX5 expression, ram sperm, sodium selenite, sperm quality

## Abstract

Cryopreservation of ram semen is an essential tool in assisted reproductive technology; however, oxidative stress generated during the freezing process may compromise sperm quality. This study evaluated the effects of Se and SeNPs on post-thaw sperm quality, PRDX5 expression, and oxidative status in cryopreserved ram semen. In this study, semen samples collected from five mature rams (three collections at 2-week intervals, yielding a total of 15 ejaculates) were frozen in liquid nitrogen using extenders supplemented with selenium (1 μg/mL, S1; 10 μg/mL, S2) or selenium nanoparticles (SeNPs; 1 μg/mL, N1; 2 μg/mL, N2) alongside a nonsupplemented control extender. Post-thaw sperm quality was evaluated using computer-assisted sperm analysis (CASA) for motility, kinematic parameters, viability, membrane integrity (HOST) assays, chromatin condensation assessment, and morphological analysis. Total oxidant status (TOS) measurements and PRDX5 gene expression analysis were performed separately. Low-dose SeNPs (1 µg/mL) significantly improved total motility (55.73 ± 19.01%), progressive motility (25.05 ± 15.34%), viability (57.27 ± 19.30%), HOST-positive spermatozoa (50.87 ± 18.91%), and morphologically normal spermatozoa (88.27 ± 4.10%) compared with the control and high-dose sodium selenite groups (*p* < 0.05). Chromatin condensation abnormalities were lowest in the SeNP-treated group. S1 and N2 also improved motility and morphology compared with the control; however, the increases were numerically smaller than those observed in the N1 group. In contrast, S2 supplementation showed limited benefit, with values that were similar to those of the control. Morphologically normal spermatozoa were highest in N1, followed by S1 and N2, while S2 and the control exhibited the lowest values (*p* < 0.05). In contrast, no significant differences were detected in TOS or PRDX5 gene expression among the experimental groups (*p* > 0.05). These findings indicate that low-dose SeNPs enhance post-thaw sperm functional integrity and cryotolerance without inducing measurable changes in bulk oxidative markers or gene transcription.

## Simple Summary

The freezing and thawing process of ram semen can damage sperm cells and reduce their fertility. Antioxidants are added to semen extenders to help protect sperm during this process. In this study, we compared the effects of different doses of conventional selenium and selenium nanoparticles on semen freezing. Our results showed that a low dose of selenium nanoparticles provided the best protection after thawing. These findings suggest that adding a low dose of selenium nanoparticles to semen extenders is a simple and effective way to improve the quality of frozen–thawed ram semen.

## 1. Introduction

Sperm cryopreservation plays a critical role in artificial insemination technologies, especially in small ruminant production, which allows the genetic potential of ram semen to be transferred to wider populations. The success of the cryopreservation process is directly related to the sperm count and functional quality obtained after thawing, which are key determinants of fertilization efficiency [1,2]. Several stress factors affect sperm cells during the freezing process. Among these factors, oxidative stress is a major contributor and develops due to the disruption of the balance between reactive oxygen species (ROS) and cellular antioxidant defense mechanisms [3].

Spermatozoa produce ROS during their metabolic activities. Reactive oxygen species normally play a role in physiological processes such as cellular signaling, capacitation, and acrosome reaction [1,3]. However, the increase in ROS production during cryopreservation leads to lipid peroxidation of the sperm plasmalemma and disruption of membrane integrity. This negative effect stems from the susceptibility of spermatozoa membranes, which have a high polyunsaturated fatty acid content, to oxidative stress [4,5]. Lipid peroxidation impairs membrane fluidity and can severely reduce sperm motility, membrane stability, and fertilization capacity [4]. Furthermore, elevated ROS levels can lead to mitochondrial dysfunction and disruption of DNA integrity, further worsening sperm function, which is associated with post-thaw quality loss and fertilization failure [4]. For these reasons, controlling oxidative stress and developing antioxidant strategies during ram sperm cryopreservation are of great importance in preserving sperm quality and improving artificial insemination success [3].

Although remarkable progress has been achieved in sperm cryopreservation techniques and in the formulation of novel extenders, it is well established that nearly 40–50% of spermatozoa experience a decline in fertilization potential following the freeze–thaw cycle [6]. Recent research indicates that supplementing semen with hydrophilic or lipophilic antioxidants, both in human and animal andrology, provides protective effects by preserving the ultrastructure and functional competence of spermatozoa [7,8]. To counteract the excessive ROS availability to preserve post-thaw sperm function, supplementing natural antioxidants to semen extenders has become common [8]. In this context, selenium (Se), an essential element involved in cellular redox regulation, has attracted particular attention due to its role in antioxidant defense mechanisms and protection against ROS-induced lipid peroxidation. Accordingly, Se supplementation has been proposed as a promising strategy to mitigate oxidative damage during cryopreservation and improve post-thaw sperm quality [9].

Selenium (Se) is a naturally occurring essential trace element involved in cellular development, apoptosis regulation, and various signaling pathways. Se protects spermatozoa from oxidative damage, and its beneficial effects on reproductive physiology have been extensively documented [10,11]. Selenium exerts its antioxidant functions primarily by modulating selenoprotein expression, whereby sulfur atoms in proteins are substituted with seleno-amino acids (e.g., L-selenomethionine, L-selenocysteine). These modifications contribute to the activity of selenoenzymes, such as glutathione peroxidases (GPxs), which are critical in protecting sperm cells from oxidative stress [12].

Despite these advantages, Se utilization is limited by concerns regarding bioavailability and potential toxicity [13]. Therefore, recent research has focused on identifying less toxic and more bioavailable forms of Se [14]. In this context, nanoparticles provide unique physicochemical properties, such as increased surface area, enhanced cellular uptake, and improved reactivity that facilitate their interaction with biological systems. Numerous studies have shown that drug absorption and bioavailability are significantly improved when delivered via nanoparticle formulations [15]. These nanoscale features have been applied to cryopreservation protocols, where they enable smaller doses to enhance antioxidant efficiency compared with conventional compounds [16].

Selenium nanoparticles (SeNPs) in particular have been described as low-toxicity agents that markedly enhance the synthesis of selenoproteins in the body [17]. Their application has attracted increasing attention in reproductive biotechnology, with several studies evaluating their antioxidant potential in improving sperm quality and fertility outcomes. In vitro supplementation with SeNPs reduces oxidative stress and positively influences sperm motility, viability, and fertilization capacity in different species, including bulls [18], rams [8,19], dogs [11,20], roosters [21], and camels [12].

Peroxiredoxins (PRDXs) are widely distributed antioxidant enzymes that play multiple roles across diverse organisms, including detoxification, immune modulation, intracellular signaling, cell proliferation, apoptosis, differentiation, and protection against oxidative damage. These enzymes represent one of the oldest families of peroxidases, characterized by their dependence on sulfhydryl groups rather than Se or heme cofactors. They are highly expressed from prokaryotic to eukaryotic systems [22,23].

Structurally, PRDXs contain one or more conserved cysteine (Cys) residues. Based on the number and positioning of these Cys residues in their catalytic domains, they are classified into six distinct isoenzymes. Different PRDX isoforms are associated with distinct biological processes. Among these isoenzymes, PRDX5 represents a thioredoxin-dependent peroxidase that has attracted particular attention due to its high expression in testicular tissue and spermatozoa, suggesting a specific role in male reproductive physiology. PRDX5 protects cellular components against oxidative stress and contributes to the maintenance of redox homeostasis in cells exposed to increased ROS levels. Since ROS negatively affects male fertility, PRDX5 may contribute to the maintenance of sperm function and genome integrity under oxidative stress [22,23,24].

In this context, we specifically focused on PRDX5 because its elevated expression in testis and spermatozoa strongly suggests a unique role in maintaining sperm motility, DNA integrity, and overall fertilizing potential under conditions of oxidative stress. Given that spermatozoa possess limited intrinsic antioxidant defenses and are highly susceptible to ROS-induced damage, PRDX5 may represent a critical antioxidant factor involved in sperm cryotolerance [25,26]. Moreover, there is a paucity of studies investigating PRDX5 expression in relation to oxidative stress or cryopreservation conditions in ram semen, underscoring the novelty and relevance of the present study.

Recent advances in nanotechnology have enabled the development of SeNPs with enhanced bioavailability, reduced toxicity, and improved antioxidant efficiency compared with conventional Se sources. Despite increasing evidence supporting their beneficial effects on sperm cryopreservation, limited information is available regarding their influence on oxidative conditions and PRDX5 expression in ram semen. Therefore, the objective of the present study was to evaluate the effects of Se and SeNPs at different concentrations on post-thaw sperm quality and PRDX5 gene expression in cryopreserved ram semen, with total oxidant status included as a complementary oxidative parameter. We hypothesized that low-dose SeNPs would enhance sperm functional parameters and cryotolerance more effectively than inorganic Se.

## 2. Materials and Methods

### 2.1. Animals and Semen Collection

This study was conducted at the University of Ondokuz Mayis in Samsun/Türkiye (northern latitude: 41.37°, eastern longitude: 36.21°) during the non-breeding season (from April to June).

In this study, we used five sexually mature male Bafra rams (*Ovis aries*) (average age 56.3 ± 4.81 months). Rams received a diet containing 400 g of hay cubes twice a day. Mineralized salt licks and clean tap water were available ad libitum. All experimental procedures were approved by the Ethics Committee for Laboratory Animals of Ondokuz Mayıs University (approval no. E-68489742-604.01-2500219665). Rams were subjected to a sexual rest period of at least 3 days prior to semen collection, and no sedation or anesthesia was applied during the electroejaculation procedure. Collections were performed three times at 2-week intervals, yielding a total of 15 ejaculates. Only one ejaculate was collected from each ram per collection day. The animals were kept under standardized feeding and housing conditions throughout the study. Semen samples were collected by electroejaculation. For semen retrieval, a specially designed electro-ejaculator was employed, consisting of a rectal probe (approximately 25 cm in length and 2.5 cm in diameter) equipped with three longitudinal electrodes connected to a voltage-regulated power supply. Before insertion, both the probe and the rectal sphincter were lubricated to facilitate placement. Electrical stimulation was then applied in cycles of 5–10 s of current exposure followed by 10 s of rest. The procedure was initiated at 4 V and gradually increased to 8 V if necessary. Semen was collected into pre-warmed tubes and immediately transferred to the laboratory in a water bath maintained at 37 °C. The interval between semen collection and the initial laboratory handling did not exceed 10 min. Each ejaculate was handled separately. Seminal plasma was not removed prior to semen processing. To minimize individual animal variation, only ejaculates meeting the following quality standards were included in the trial: ejaculate volume between 0.5–2 mL, sperm concentration ≥ 3 × 10^9^ spermatozoa/mL, progressive motility > 80%, and <10% abnormal spermatozoa.

### 2.2. Extender Preparation and Semen Processing

All chemicals used for extender preparation were of analytical grade and obtained from Sigma-Aldrich^®^ (St. Louis, MO, USA), unless otherwise specified; nanoparticle Se was purchased from Nanocs Inc.^®^ (New York, NY, USA) with a reported particle size of approximately 100 nm, according to the manufacturer. The semen extender was formulated by dissolving Tris (0.2975 M), citric acid (0.1053 M), fructose (0.0826 M), penicillin (100,000 IU), and streptomycin (100 mg) in 100 mL of distilled water. To this Tris base solution, 6 mL of glycerol and 20 mL of egg yolk were added to 74 mL of the prepared extender. The final diluent was then divided into five treatment groups. The control group received no supplementation, while the experimental groups included 1 µg/mL nanoparticle Se (N1), 2 µg/mL nanoparticle Se (N2), 1 µg/mL sodium selenite (S1), and 10 µg/mL sodium selenite (S2) [8,27,28]. Each ejaculate was portioned into five equal parts and extended at 37 °C to obtain a final sperm concentration from 1.0–1.5 × 10^9^ spermatozoa/mL. The semen samples were cooled stepwise by placing them in a refrigerator, allowing a gradual decrease from 37 °C to 5 °C, followed by an equilibration period of 4 h at 5 °C. After equilibration at 5 °C for 4 h, semen aliquots were loaded into 0.25-mL French straws and sealed. Straw filling and sealing procedures were performed under controlled temperature conditions at 5 °C. The straws were placed horizontally on a rack 4 cm above liquid nitrogen vapor for 10 min to allow gradual cooling to about −120 °C, then plunged into liquid nitrogen [29]. After this phase, the straws were plunged into liquid nitrogen (−196 °C) for long-term storage.

### 2.3. Assessment of Semen

Semen samples were thawed in a water bath at 37 °C for 30 s and assessed for motility, kinematic traits, viability, plasma membrane integrity, morphological abnormalities, chromatin condensation, total oxidant Status (TOS), and PRDX5 gene expression. All post-thaw evaluations were initiated immediately after thawing to minimize temperature and time-related variability.

### 2.4. Assessment of Sperm Motility and Kinematic Parameters

Sperm motility and kinematic characteristics were evaluated post-thaw using a Computer-Assisted Sperm Analysis system (CASA; Sperm Class Analyzer (SCA), Version 6.5.0.91, Microptic, Barcelona, Spain). Analyses were performed under a phase-contrast microscope (Eclipse, Nikon, Tokyo, Japan) equipped with a 10× objective and a high-speed camera operating at 60 frames per second, with the stage temperature maintained at 37 °C. Sperm images were captured using a high-frame-rate Basler digital camera supplied by the CASA system manufacturer (Microptic S.L., Barcelona, Spain) mounted on a phase-contrast microscope. CASA settings were standardized for small ruminant spermatozoa as follows: frame rate 25 Hz, acquisition time 1 s, minimum contrast 80, minimum cell size 4 µm^2^, VAP cut-off 20 µm/s, and VSL cut-off 10 µm/s. At least 200 spermatozoa from a minimum of five randomly selected microscopic fields were analyzed per sample. The CASA system quantified total motility (TM, %) and progressive motility (PM, %) alongside kinematic variables, including average path velocity (VAP, µm/s), straight-line velocity (VSL, µm/s), curvilinear velocity (VCL, µm/s), linearity (LIN, %), straightness (STR, %), and amplitude of lateral head displacement (ALH, µm). In addition, total sperm concentration was adjusted to the working range recommended for CASA analysis prior to evaluation.

### 2.5. Assessment of Sperm Viability

Sperm cell viability was determined using the Eosin–Nigrosin staining technique (2% Eosin prepared in 3% sodium citrate). For this procedure, 10 μL of thawed semen was placed on a pre-warmed slide and mixed with 20 μL of the Eosin–Nigrosin solution. A thin smear was prepared and examined under a phase-contrast microscope with a heated stage maintained at 37 °C. A total of 200 spermatozoa were evaluated under a 20× objective lens across different microscopic fields. Spermatozoa that absorbed the stain and appeared pink or red were classified as non-viable, whereas unstained cells were considered viable. Results were expressed as the percentage of viable spermatozoa relative to the total number of spermatozoa counted.

### 2.6. Assessment of Plasma Membrane Integrity

Plasma membrane integrity was determined using the hypo-osmotic swelling test (HOST). For each assessment, 100 μL of HOST solution prepared by dissolving 1.351 g of fructose and 0.735 g of sodium citrate was placed in an Eppendorf tube, into which 10 μL of thawed semen was added. The mixture was incubated at 37 °C for 1 h. Following incubation, 10 μL of the suspension was smeared onto a glass slide and left to air dry at a 45° angle. Once dried, 200 spermatozoa were examined under a microscope at 40× magnification. Cells displaying characteristic swelling of the head and coiling of the tail were considered to have intact plasma membranes, while non-reactive spermatozoa were classified as membrane-damaged.

### 2.7. Assessment of Sperm Morphology

Sperm morphological abnormalities were analyzed using the morphology module of the CASA system in combination with the SpermBlue^®^ staining kit (Microptic S.L., Barcelona, Spain). Following freeze–thaw procedures, a 10 μL aliquot of semen was smeared onto a glass slide and air-dried. The dried smears were immersed in SpermBlue stain for 2 min at room temperature, then rinsed twice with distilled water and allowed to dry again. Subsequently, at least 200 spermatozoa were evaluated morphologically with the CASA system. The abnormalities were classified involving the head, midpiece, and tail, and the overall percentage of defective spermatozoa was recorded. Head abnormalities included defects such as abnormal head shape and size, while midpiece abnormalities comprised bending or thickening, and tail abnormalities included coiled, bent, or broken tails.

### 2.8. Assessment of Sperm Chromatin Condensation

Chromatin condensation of spermatozoa was evaluated using the Toluidine Blue (TB) staining technique [30]. For this procedure, 10 μL of thawed semen was smeared onto glass slides and air-dried. The smears were fixed in ethanol-acetic acid solution (3:1, *v*/*v*) for 1 min, followed by immersion in 70% ethanol for 3 min. Samples were then hydrolyzed in 4N hydrochloric acid for 25 min, rinsed with distilled water, and dried at room temperature. After drying, each slide was treated with 30 μL of 0.025% (*w*/*v*) TB solution prepared in McIlvaine buffer, covered with a coverslip, and left for 3 min. Stained preparations were examined under a light microscope (Eclipse, Nikon, Tokyo, Japan) at 100× magnification. Spermatozoa with intact chromatin appeared light blue and were considered normal, whereas cells exhibiting damaged chromatin stained dark blue to purple and were classified as abnormal. Results were expressed as the percentage of abnormal spermatozoa compared with the total number of spermatozoa evaluated, based on the assessment of 200 spermatozoa per sample.

### 2.9. Determination of Total Oxidant Status

The total oxidant capacity of semen samples was measured using a commercial Total Oxidant Status (TOS) ELISA kit (Rel Assay Diagnostic, Gaziantep, Türkiye; Total Oxidant Status Assay Kit^®^). The kit was stored at 4 °C until the day of analysis, and all reagents were brought to room temperature before use. Semen samples were processed according to the manufacturer’s instructions. Thawed samples at 37 °C were centrifuged at 800× *g* for 10 min, and 30 μL of the resulting supernatant was transferred to an Eppendorf tube. Subsequently, 200 μL of Reagent 1 from the kit was added to the sample and mixed thoroughly using a vortex. The mixture was then transferred to a flat-bottom microplate for measurement. The microplate was initially read using an ELISA reader (Thermo MULTISKAN EX, spectrophotometer, Thermo Fisher Scientific, Vanta, Finland) at 530 nm to obtain the first absorbance value (A1). Following this, 10 μL of Reagent 2 was added to each well, mixed with a vortex, and incubated at room temperature for 10 min. After incubation, the second absorbance reading (A2) was recorded at 530 nm. All samples were measured in duplicate, and the mean absorbance value was used for TOS calculation. The TOS values were calculated using the following formula:

Δabs = A2 − A1 (standard or sample)

TOS (μmol/L) = Δabs Sample/Δabs Standard × Standard concentration (10 μmol/L)

All measurements were expressed in μmol/L.

### 2.10. RNA Extraction and cDNA Synthesis

Total RNA was isolated from both fresh and frozen-thawed semen samples using TRIzol reagent (Invitrogen, Carlsbad, CA, USA) in accordance with the manufacturer’s instructions. The purity and concentration of the extracted RNA were determined spectrophotometrically with a NanoDrop 2000 instrument (Thermo Scientific, Wilmington, DE, USA). RNA aliquots were stored at –80 °C until further use. Complementary DNA (cDNA) was synthesized using the iScript™ cDNA Synthesis Kit (Bio-Rad, Hercules, CA USA), following the manufacturer’s instructions. This kit employs a combination of oligo(dT) and random primers to optimize reverse transcription efficiency. For each reaction, 4 μL of 5× iScript Reverse Transcriptase, 5 μL of nuclease-free water, and 2 μL of RNA were combined in a 0.2-mL PCR tube. The thermal protocol involved incubation at 25 °C for 5 min, 46 °C for 20 min, and 95 °C for 1 min. An RNA-free negative control was included to check for contamination. Following cDNA synthesis, samples were immediately placed on ice and stored at –80 °C until quantitative analysis.

### 2.11. Primer Design

Gene-specific primers targeting the PRDX5 gene (NCBI Reference Sequence: XM_004019653.5) were designed to assess mRNA expression levels. The β-actin gene (NCBI Reference Sequence: NM_001009784.1) served as the internal reference for normalization of expression data [20]. All primers were based on the respective gene sequences and were synthesized commercially (Table 1).

### 2.12. Reverse Transcription Quantitative PCR Analysis

Quantitative real-time PCR (qPCR) was conducted to accurately measure the mRNA expression levels of the PRDX5 gene. The β-actin gene was employed as an internal reference for normalization. Reverse transcription quantitative PCR (RT-qPCR) reactions were performed using the Bio-Rad SsoAdvanced Universal Inhibitor-Tolerant SYBR Green Supermix Kit according to the manufacturer’s instructions. Each 20-μL reaction contained 10 μL of SYBR Green Supermix, 1 μL each of forward and reverse primers, 6 μL of nuclease-free water, and 2 μL of cDNA template. Amplifications were performed on a Bio-Rad CFX96 Real-Time PCR system, with all samples analyzed in triplicate alongside a no-template control. The threshold cycle (Ct) values for PRDX5 were normalized to β-actin, and relative expression levels were calculated using the 2−ΔΔCt method [31].

### 2.13. Statistical Analyses

For normality analyses of quantitative data, the Shapiro–Wilk test was used when *n* was <50, and the Kolmogorov–Smirnov test was used when *n* was ≥50. One-way analysis of variance (ANOVA) was used for between-group comparisons of normally distributed data. Homogeneity of variance was assessed with the Levene test, and the Welch correction was applied when the assumption of homogeneity of variance was not met. For multiple comparison analyses of parametric tests, the Tukey HSD test was used when variances were homogeneous, and the Tamhane T2 test was used when variances were not homogeneous. For between-group comparisons of non-normally distributed data, the Kruskal–Wallis H test was used. In cases where significant differences were found using the Kruskal–Wallis H test, the Dunn test with Bonferroni correction was used for multiple comparison analyses. Correlation analysis was used to evaluate relationships between quantitative data, and the findings of the correlation analysis were presented graphically. The Pearson correlation coefficient was calculated for relationships between normally distributed data. The Spearman correlation coefficient was used for data that were not normally distributed. Correlation analysis was performed using Spearman’s rank correlation test, and the results were visualized as a correlation matrix in which color intensity and circle size reflect the direction and strength of the correlations. The R program [32], (version 4.2.2; R Foundation for Statistical Computing, Vienna, Austria), along with the corrplot (version 0.92) and psych (version 2.3.3) packages [33,34] were used for the analyses. Statistical analyses were evaluated at a significance level of *p* < 0.05.

## 3. Results

Post-thaw sperm motility and kinematic parameters are presented in Table 2. A statistically significant difference was observed among the experimental groups for total motility (*p* < 0.001). The N1 group exhibited significantly higher total motility compared with the control and S2 groups (*p* < 0.05). The S1 and N2 groups showed intermediate values and did not differ significantly from N1. The lowest total motility values were observed in the control and S2 groups, which did not differ significantly from each other. Progressive motility also differed significantly among groups (*p* = 0.008). The N1 group had significantly higher progressive motility than the control group (*p* < 0.05). No significant differences were observed between N1 and S1 or N2, while the S2 group did not differ significantly from the control. No statistically significant differences were detected among groups for sperm concentration or kinematic parameters, including curvilinear velocity (VCL), average path velocity (VAP), straight-line velocity (VSL), straightness (STR), linearity (LIN), and amplitude of lateral head displacement (ALH) (*p* > 0.05 for all). Given the sensitivity of CASA-derived motility and kinematic parameters to factors such as sperm concentration and tracking conditions, these results should be interpreted with appropriate caution.

The results for sperm viability, plasma membrane integrity (HOST), chromatin condensation, and morphological parameters are summarized in Table 3. A significant difference was detected among groups for HOST-positive spermatozoa (*p* = 0.035). Groups N1, N2, S1, and S2 showed significantly higher rates of HOST-positive cells than the control group (*p* < 0.05). The control group showed the lowest membrane integrity, while there was no statistically significant difference between the other groups.

Sperm viability also differed significantly among groups (*p* = 0.005). The highest viability was observed in the N1 group, which was significantly higher than that of the control and S2 groups (*p* < 0.05). The S1 and N2 groups did not differ significantly from N1.

Chromatin condensation abnormalities assessed by TB staining showed a highly significant group effect (*p* < 0.001). The N1 group exhibited the lowest percentage of TB-positive spermatozoa, indicating superior chromatin integrity compared with all other groups (*p* < 0.05). The highest levels of chromatin condensation abnormalities were observed in the control and S2 groups, which did not differ significantly from each other.

Significant differences were observed among groups for head abnormalities (*p* < 0.001) and tail abnormalities (*p* < 0.001), whereas midpiece abnormalities did not differ significantly among groups. The control group exhibited the highest incidence of head and tail abnormalities, while the N1 group had significantly lower head and tail defect rates than the control and S2 groups (*p* < 0.05). The S1 and N2 groups had intermediate values. The percentage of morphologically normal spermatozoa differed significantly among groups (*p* < 0.001). The N1 group had the highest proportion of morphologically normal spermatozoa, which was significantly greater than that of the control and S2 groups (*p* < 0.05).

The relative PRDX5 gene expression levels and TOS values are presented in Table 4. No statistically significant differences were observed among the experimental groups for PRDX5 expression (*p* = 0.802). Although the S2 group had the highest mean expression value, the large intersample variability precluded statistical significance.

Similarly, no significant differences were detected among groups for TOS levels (*p* = 0.331). The lowest mean TOS value was observed in the N1 group; however, this reduction was not statistically significant compared with the other groups. Given the considerable intragroup variability observed in these assays, the results should be interpreted with appropriate caution.

**Figure 1:**
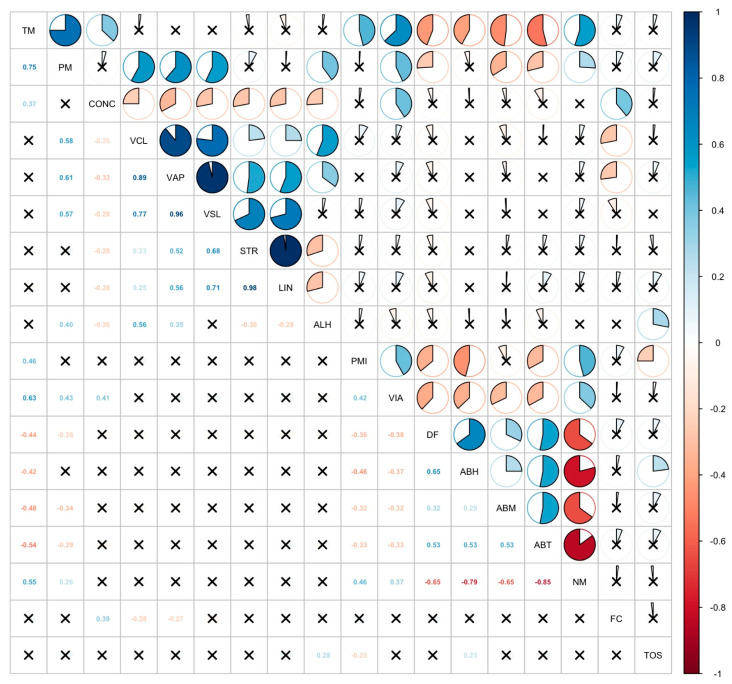
Significant correlations between spermatological parameters and PRDX5 gene expression levels in study groups. Correlation matrix illustrating the relationships between spermatological, functional, and oxidative parameters. Each cell represents the Spearman correlation coefficient (r) between two variables. Blue color tones indicate positive correlations, whereas red color tones indicate negative correlations. The intensity of the color and the proportion of the filled circle correspond to the magnitude of the correlation coefficient. Numerical values within the cells indicate the correlation coefficients (r). Cells marked with an “x” indicate non-significant correlations (*p* > 0.05). TM: Total Motility; PM: Progressive Motility; Conc: Concentration; VCL: Curvilinear Line Velocity; VAP: Average Path Velocity; VSL: Straight Line Velocity; STR: Straightness; LIN: Linearity; ALH: Amplitude of the Lateral Head; PMI: Plasma Membrane Integrity; VIA: Viability; DF: DNA Fragmentation; ABH: Head Abnormality; ABM: Midpiece Abnormality; ABT: Tail Abnormality; NM: Normal Morphology; FC: Fold Change; TOS: Total Oxidant Status.

The results of the correlation analysis are presented in Figure 1. This figure is presented for illustrative purposes to support the overall interpretation of the data. Multiple statistically significant associations were identified among spermatological parameters. Total motility (TM) exhibited strong positive correlations with progressive motility (PM) (r = 0.75), curvilinear velocity (VCL) (r = 0.58), average path velocity (VAP) (r = 0.89), and straight-line velocity (VSL) (r = 0.77). Similarly, PM was strongly correlated with VCL (r = 0.61), VAP (r = 0.77), and VSL (r = 0.96).

Kinematic parameters also showed close interrelationships. Strong positive correlations were found between linearity (LIN) and straightness (STR) (r = 0.98), as well as between LIN and wobble (WOB) (r = 0.94). In addition, VAP was highly associated with VCL (r = 0.90) and VSL (r = 0.92).

Negative associations were detected between morphological abnormalities and motility-related parameters. Tail abnormalities (ABT) were negatively correlated with TM (r = −0.54), PM (r = −0.48), VCL (r = −0.44), and VSL (r = −0.29), indicating that increased tail defects impair sperm motility. Similarly, abnormal head morphology (ABH) was positively associated with DNA fragmentation (DF) (r = 0.65), while ABT was negatively correlated with normal morphology (NM) (r = −0.79).

Collectively, these results highlight that improvements in motility are closely linked with enhanced velocity-related parameters, whereas higher morphological abnormalities correspond to reduced motility and structural integrity.

## 4. Discussion

The present study examined the effects of Se and SeNPs on the quality of cryopreserved ram semen, with a comprehensive evaluation of motility, viability, plasma membrane integrity, morphology, chromatin condensation, oxidative status, and PRDX5 gene expression. The significant finding was that supplementation with low-dose SeNPs (1 μg/mL, N1) provided the most consistent protective effects, enhancing post-thaw spermatological parameters without significantly altering TOS or PRDX5 gene expression. Importantly, the absence of significant changes in oxidative markers indicates that the observed functional improvements should not be interpreted as direct evidence of a measurable antioxidant effect.

Sperm motility is widely recognized as a key determinant of fertilization potential. In this study, total motility was significantly higher in the N1 group than in the control and S2 groups, whereas progressive motility was significantly increased in the N1 group only when compared with the control group. These improvements corroborate previous work in bulls, where SeNPs preserved motility after cryopreservation more effectively than inorganic Se forms [18]. Similar benefits have been reported in roosters and camels [12,21], suggesting that SeNPs exert cross-species protective effects. The nanoscale size of SeNPs allows for superior cellular uptake and bioavailability compared with sodium selenite, resulting in improved antioxidant capacity within sperm cells [14,17]. Mechanistically, enhanced motility may be attributed to the stabilization of mitochondrial function. Mitochondria supply ATP for sperm motility, and oxidative stress during freezing can impair mitochondrial membrane potential [35]. By scavenging ROS, SeNPs likely preserve mitochondrial integrity, enabling sustained ATP synthesis and flagellar activity. The observed dose-dependent effects, where low-dose SeNPs were beneficial but high-dose sodium selenite (S2) was less effective, highlight the importance of precise dosing to balance antioxidant protection and avoid pro-oxidant toxicity [13]. No significant differences were detected in specific CASA-derived kinematic traits (VCL, VSL, VAP, STR, LIN, and ALH). This suggests that SeNPs primarily increased the proportion of motile and progressively motile sperm rather than altering the trajectory characteristics of individual spermatozoa. Previous studies also showed that antioxidant supplementation improves the number of motile sperm without necessarily changing detailed motion kinematics [7]. Maintaining a higher percentage of motile cells may be more critical for fertilization success than fine-tuning individual kinematic profiles, especially in artificial insemination protocols. However, given the lack of significant changes in oxidative markers and the variability observed in CASA parameters, these interpretations should be considered exploratory rather than definitive.

Viability is another essential predictor of sperm fertilization capacity. In the present study, eosin-nigrosin staining revealed significantly higher viability in SeNP-supplemented group N1 than in the S1, N2, S2, and control groups. Improved viability is likely mediated by the reduction in lipid peroxidation, which otherwise compromises plasma membrane integrity. The high proportion of polyunsaturated fatty acids in ram sperm membranes renders them highly susceptible to ROS-induced peroxidation [10]. SeNPs act through the activation of selenoproteins, such as glutathione peroxidases, which detoxify hydrogen peroxide and lipid hydroperoxides, thereby preventing structural disruption of the sperm membrane [10].

The HOST further confirmed superior membrane functionality in the N1 group. HOST-positive cells reflect intact membranes capable of responding to osmotic stress, and higher percentages indicate better resilience against cryodamage. Comparable improvements in HOST values have been reported in buffalo bull semen supplemented with Se [36] and rooster semen treated with SeNPs [21]. These results suggest that SeNPs provide broad-spectrum membrane protection, potentially through both antioxidant effects and stabilization of membrane phospholipids.

One of the most striking outcomes was reduced chromatin condensation abnormalities in the Se and SeNP groups, especially N1. Toluidine Blue staining indicated that SeNPs significantly decreased DNA fragmentation compared with the control and S2 groups. This is consistent with findings in human andrology, where Se supplementation reduced oxidative DNA damage in asthenoteratozoospermic patients [19]. In rams, dietary Se supplementation improved sperm DNA integrity and reduced chromatin abnormalities, indicating a protective role against oxidative stress during spermatogenesis [37]. Experimental models revealed that green-synthesized SeNPs significantly reduced sperm DNA fragmentation in streptozotocin-induced diabetic mice, as assessed by the comet assay [38]. Oxidative stress induces single- and double-strand DNA breaks, base modifications, and chromatin crosslinking [39,40]. By scavenging ROS and enhancing antioxidant enzyme activity, SeNPs protect sperm chromatin, thereby preserving genomic integrity critical for fertilization and embryo development. The protective role of SeNPs on chromatin stability may also involve indirect regulation of protamination during spermatogenesis, though this hypothesis requires further validation in vivo. In livestock breeding, reducing DNA fragmentation is highly desirable since sperm DNA damage is associated with reduced conception rates, impaired embryonic development, and increased miscarriage risk [41].

Morphological assessment revealed significant reductions in head and tail defects in SeNP groups, while midpiece abnormalities remained unchanged. The N1 group had the highest proportion of morphologically normal sperm, which was considerably greater than that of the control. These findings are consistent with prior reports indicating that antioxidants reduce cryo-injury-related morphological defects [40]. The persistence of midpiece abnormalities may reflect either inherent susceptibility differences across sperm compartments or limitations in detection sensitivity. Nonetheless, the strong negative correlation between tail abnormalities and motility underscores the functional significance of preserving morphology. Reduced head abnormalities are significant, as head defects are strongly linked to DNA fragmentation. The present study confirmed a positive correlation between abnormal heads and chromatin condensation defects, suggesting that SeNPs protect the nuclear and structural components of spermatozoa. Accordingly, the morphological findings in the present study should be regarded as supportive observations rather than primary indicators of antioxidant effects.

Contrary to expectations, TOS values did not differ significantly among groups, although the lowest mean values were recorded in the N1 group. Several explanations may account for this discrepancy. First, TOS measures total oxidant load in bulk semen samples and may not capture localized intracellular ROS dynamics. Second, spermatozoa possess limited intrinsic antioxidant defenses, meaning that exogenous supplementation may exert protective effects at the cellular level without altering overall oxidant concentrations measurable in seminal plasma [42]. Despite the absence of significant TOS changes, functional improvements in motility, viability, and morphology clearly indicate that SeNPs mitigated oxidative stress at the cellular level. These findings are consistent with a previous study [7], where curcumin nanoparticles improved bovine semen quality without significant changes in bulk oxidative markers. Therefore, functional sperm quality parameters may serve as more sensitive indicators of cryoprotection than global oxidative assays.

Another notable result was the absence of significant differences in PRDX5 expression among groups. PRDX5 is an important antioxidant enzyme highly expressed in the testis and spermatozoa, with roles in neutralizing hydrogen peroxide and regulating redox signaling [22,43].

However, spermatozoa are mature cells and are largely considered transcriptionally and translationally quiescent; this limits their capacity for changes in gene expression after thawing. Therefore, it is thought that PRDX5 activity is modulated at the protein level or through post-translational regulatory mechanisms rather than through gene-level changes in the post-thawing period [44,45].

Selenium, as an essential cofactor of antioxidant enzymes, including glutathione peroxidases and thioredoxin reductases, may exert its protective effects primarily through enhancing protein activity and redox balance rather than altering gene transcription. Both sodium selenite and SeNPs can scavenge ROS and support enzymatic antioxidant defenses, although their mechanisms may differ. Selenium nanoparticles, due to their higher bioavailability and cellular uptake, could enhance intracellular selenoprotein activity more efficiently than inorganic Se forms, thereby improving sperm function without necessarily changing PRDX5 mRNA levels. This aligns with previous findings [46] where SeNPs elevated glutathione peroxidase activity in seminal fluid, suggesting enhanced antioxidant capacity without significant changes in gene expression.

Contrary to expectations, TOS values did not differ significantly among the experimental groups, although the lowest mean values were observed in the N1 group. This apparent discrepancy can be explained by the intrinsic limitations of TOS, which reflects the overall oxidant load in bulk semen samples and may fail to capture localized intracellular ROS dynamics. Cryopreservation is known to induce rapid and spatially restricted ROS generation, particularly within mitochondria, as a consequence of electron transport chain disruption, osmotic stress, and membrane phase transitions during freezing and thawing. Importantly, such mitochondrial- and membrane-associated ROS production may impair sperm function without necessarily leading to detectable alterations in global oxidative markers measured in the extracellular milieu [47,48]. Consistent with this concept, previous studies have demonstrated that improvements in post-thaw sperm motility, viability, and membrane integrity following antioxidant or extender supplementation can occur independently of significant changes in bulk oxidative stress indices, supporting the notion that functional sperm parameters may represent more sensitive indicators of cryoprotection than global oxidant assays [49,50]. In the present study, despite the absence of significant differences in TOS values, the observed enhancements in sperm functional characteristics clearly suggest that SeNP supplementation mitigated oxidative stress at the cellular and subcellular levels.

Another important observation was the lack of significant differences in PRDX5 expression among groups. PRDX5 is a key intracellular antioxidant enzyme abundantly expressed in testicular tissue and spermatozoa, where it plays a critical role in detoxifying hydrogen peroxide and regulating redox-sensitive signaling pathways [51,52]. However, mature spermatozoa are transcriptionally and translationally quiescent cells, limiting the potential for post-thaw alterations in gene expression. Therefore, unchanged PRDX5 mRNA levels do not necessarily indicate an absence of antioxidant response but may instead reflect a stable transcriptional baseline. It is plausible that PRDX5 activity is modulated predominantly at the protein or post-translational level, as previously demonstrated in human and boar spermatozoa [25]. Selenium, as an essential component of several antioxidant enzymes, including glutathione peroxidases and thioredoxin reductases, may exert its protective effects primarily by enhancing enzymatic activity and maintaining intracellular redox balance rather than by altering gene transcription. Both sodium selenite and SeNPs can scavenge ROS; however, their modes of action may differ substantially. Due to their enhanced bioavailability and cellular uptake, SeNPs may more efficiently support intracellular selenoprotein function, thereby improving sperm cryotolerance without necessarily inducing measurable changes in PRDX5 transcript levels. This interpretation is consistent with previous findings [46], where increased glutathione peroxidase activity in seminal fluid following SeNP supplementation was observed despite minimal changes in gene-expression profiles. Consequently, reliance solely on transcript-level analyses may underestimate the true antioxidative and cytoprotective impact of SeNPs.

Taken together, these findings suggest that the beneficial effects of the N1 extender on sperm cryotolerance are more likely mediated through modulation of intracellular ROS distribution, preservation of mitochondrial integrity, and stabilization of redox-sensitive signaling pathways rather than through a measurable reduction in total oxidant load.

A notable limitation of the present study is the lack of direct assessment of intracellular ROS production during the cryopreservation process. Although TOS provides useful information regarding overall oxidative balance, it does not allow for the detection of transient or compartment-specific ROS generation at the subcellular level. In addition, although the beneficial effects of SeNPs observed in this study suggest a potential role in preserving mitochondrial integrity during cryopreservation, direct evaluation of mitochondrial activity was not performed. Parameters such as mitochondrial membrane potential, ATP production, or oxygen consumption rate could provide more definitive evidence regarding mitochondrial functionality and bioenergetic status. Furthermore, the activity of the glutathione-dependent antioxidant system, including reduced glutathione levels and GPx activity, was not assessed. Given the central role of glutathione-mediated pathways in maintaining redox homeostasis and protecting sperm mitochondria against oxidative injury, the absence of these measurements limits a more comprehensive interpretation of the underlying antioxidant mechanisms. Therefore, future studies incorporating targeted ROS assays, mitochondrial functional analyses, and glutathione-related evaluations are warranted to further elucidate the mechanistic basis of SeNP-mediated cryoprotection. The relatively high standard deviation values observed for PRDX5 expression and TOS levels compared with their respective means likely reflect inherent biological heterogeneity among ejaculates rather than methodological inconsistency. This variability may explain the absence of statistically significant differences between experimental groups. Nevertheless, such heterogeneity represents a limitation of the present study and should be addressed in future investigations using larger sample sizes.

Consequently, evaluating only transcript levels may underestimate the antioxidative impact of SeNPs. Future investigations should therefore evaluate PRDX5 protein abundance and enzymatic activity using proteomic approaches rather than relying solely on mRNA analysis. This would provide a more accurate assessment of how SeNPs influence intracellular antioxidant defense systems during cryopreservation. This approach would provide a more accurate assessment of how SeNPs influence intracellular antioxidant defense systems, as emphasized by Abedin et al. [53], who highlighted the importance of proteomic evaluations in understanding the effects of SeNPs on sperm quality.

The correlation analysis underscored the interdependence of sperm traits. Strong positive correlations were observed between total motility, progressive motility, and velocity parameters, while negative correlations linked morphological defects to motility and viability. These findings highlight that sperm structural integrity underpins functional competence, reinforcing the rationale for using morphology to predict fertility outcomes [54]. The observed associations also provide mechanistic insight, suggesting that the superior outcomes in the N1 group resulted from cumulative protection of membranes, chromatin, and morphology, which in turn supported motility and viability.

Selenium supplementation has long been recognized for its role in protecting spermatozoa against oxidative stress; however, the way Se is delivered critically influences its bioavailability and efficacy. Conventional sodium selenite (inorganic Se) exhibits antioxidant benefits at low concentrations. However, sodium selenite has a narrow safety margin and may become cytotoxic at higher doses due to reactive selenide intermediates [13]. In contrast, SeNPs provide superior bioavailability, lower toxicity, and enhanced cellular uptake due to their nanoscale dimensions and large surface area-to-volume ratio [14,17]. Comparative studies in livestock species consistently report that SeNP supplementation yields greater improvements in post-thaw sperm motility, viability, and membrane integrity than sodium selenite [8,18]. For example, in bull and ram semen, SeNPs markedly reduced morphological defects and DNA fragmentation, whereas sodium selenite at higher concentrations often failed to provide additional benefits or even produced results similar to control groups [12,19]. These findings suggest that SeNPs outperform traditional Se forms in cryoprotection and offer a safer and more efficient strategy to improve semen quality and fertility outcomes in reproductive biotechnology.

The comparison between different Se and SeNP concentrations revealed significant dose-dependent effects. While low-dose SeNPs (1 μg/mL) provided the most consistent improvement, the higher dose of sodium selenite (10 μg/mL) did not improve total motility and showed values comparable to the control group. This is consistent with the dual antioxidant-pro-oxidant nature of Se. At low concentrations, Se supports antioxidant defenses, but at higher levels, it can generate reactive selenide species that exacerbate oxidative stress [13]. These findings emphasize the necessity of carefully optimizing Se dosage in extender formulations to avoid potential cytotoxicity.

## 5. Conclusions

Low-dose SeNP supplementation significantly improved post-thaw sperm motility, viability, membrane integrity, morphology, and chromatin stability in cryopreserved ram semen, indicating enhanced cryotolerance and functional integrity of spermatozoa. In contrast, total oxidant status and PRDX5 gene expression remained unchanged, suggesting that SeNPs exert their protective effects primarily through the stabilization of sperm membranes and chromatin rather than through modulation of global oxidative markers or transcriptional regulation. Additionally, baseline (pre-freeze) measurements of TOS were not performed. Thus, ejaculate to ejaculate heterogeneity may have contributed to the variability observed in oxidative and antioxidant related endpoints. However, the present study has several limitations. Fertility outcomes were not evaluated in vivo, and therefore it remains unclear whether the observed improvements in post-thaw sperm quality translate into increased conception rates. In addition, oxidative stress was assessed solely via total oxidant status, which may not fully reflect the complexity of intracellular redox dynamics in spermatozoa. Moreover, PRDX5 evaluation was restricted to mRNA expression, whereas protein abundance and enzymatic activity would provide a more comprehensive understanding of antioxidant responses. Future studies should incorporate in vivo fertility trials, expanded oxidative stress profiling, and multi-omics approaches, including proteomics and metabolomics, to further elucidate the mechanisms underlying SeNP-mediated cryoprotection and to optimize their application in ram semen preservation.

## Figures and Tables

**Table 1:** Primer sequences for RT-qPCR analysis.

Gene	Primer Sequences (5′–3′)	Product Sizes (bp)
B-actin	F: CCCTGGAGAAGAGCTACGAG R: GGTAGTTTCGTGAATGCCGC	131
PRDX5	F: TGAGTCTGCGGTGGTTACG R: GCCAAAAGTCCAGTCGAGGA	138

**Table 2:** Comparative evaluation of sperm motility, progressive motility, concentration, and kinematic parameters of ram sperm samples in the study groups (*n* = 15 per group).

	Groups	M	SD	MED	IQR	Min	Max	Statistic	*p*-Value
Motility (%)	Control ^b^	29.00	9.35	29.79	10.89	11.49	48.02	χ^2^ = 23.831	*p* < 0.001 ^KW^
	N1 ^a^	55.73	19.01	47.27	16.94	33.73	98.25		
	N2 ^a^	40.38	16.05	39.93	23.27	17.20	77.83		
	S1 ^a^	47.09	16.35	41.73	20.09	26.13	90.86		
	S2 ^b^	34.81	14.73	33.81	19.54	17.52	68.09		
Progressive Motility (%)	Control ^b^	11.72	4.94	10.29	6.14	5.07	23.63	F = 4.168	*p* = 0.008 ^WAOV^
	N1 ^a^	25.05	15.34	17.94	18.78	8.14	59.03		
	N2 ^ab^	16.82	8.11	15.6	13.09	5.79	32.66		
	S1 ^ab^	21.69	13.02	18.13	20.08	7.11	47.33		
	S2 ^ab^	14.97	6.32	15.19	8.42	4.90	27.26		
Concentration (×10^6^/mL)	Control	18.89	12.02	14.98	14.25	4.29	41.71	χ^2^ = 1.011	*p* = 0.908 ^KW^
	N1	20.20	12.81	17.10	12.61	6.56	54.44		
	N2	22.80	13.20	19.30	15.34	2.58	51.97		
	S1	21.43	13.28	18.22	16.98	7.55	56.97		
	S2	20.07	12.63	17.10	19.65	4.10	43.98		
VCL	Control	47.93	12.59	44.46	8.17	28.54	85.45	χ^2^ = 0.899	*p* = 0.925 ^KW^
	N1	49.20	17.33	42.25	22.8	25.72	88.12		
	N2	49.57	15.87	49.94	15.45	29.31	95.71		
	S1	51.24	14.50	49.15	27.03	31.92	72.73		
	S2	46.22	18.36	47.36	12.67	5.22	79.56		
VAP	Control	30.74	8.4	30.89	11.39	16.12	45.57	χ^2^ = 0.466	*p* = 0.977 ^KW^
	N1	32.79	11.98	30.65	18.68	16.29	53.43		
	N2	32.04	13.3	31.43	14.72	16.11	69.05		
	S1	33.58	13.83	30.85	15.55	17.34	61.39		
	S2	33.21	11.17	32.36	11.51	17.91	55.61		
VSL	Control	21.41	7.48	23.41	12.82	9.70	32.63	χ^2^ = 0.525	*p* = 0.971 ^KW^
	N1	23.14	9.55	22.16	13.82	10.08	39.21		
	N2	22.13	10.69	18.83	13.09	8.63	49.63		
	S1	23.56	13.35	18.55	14.78	9.94	51.40		
	S2	23.47	9.57	22.7	15.32	10.85	38.68		
STR	Control	61.28	8.88	61.05	14.66	49.01	78.04	χ^2^ = 2.466	*p* = 0.651 ^KW^
	N1	62.21	8.05	62.90	11.21	50.28	75.18		
	N2	59.58	6.86	58.62	10.98	45.65	68.85		
	S1	60.85	9.99	63.73	14.97	45.16	74.69		
	S2	63.77	9.07	66.67	8.41	45.37	74.96		
LIN	Control	41.86	12.92	42.60	23.45	24.79	65.40	F = 0.456	*p* = 0.768 ^AOV^
	N1	43.58	10.99	45.47	15.26	27.56	62.46		
	N2	39.49	9.88	37.83	15.34	22.55	54.30		
	S1	42.09	13.17	38.79	18.32	24.30	64.04		
	S2	44.92	11.21	49.26	10.16	25.08	60.04		
ALH	Control	2.14	0.61	1.93	0.62	1.56	4.03	χ^2^ = 0.354	*p* = 0.986 ^KW^
	N1	2.22	0.63	1.96	0.43	1.67	3.58		
	N2	2.19	0.73	2.01	0.21	1.43	4.45		
	S1	2.13	0.42	2.01	0.24	1.62	3.16		
	S2	2.10	0.43	1.88	0.62	1.61	2.93		

Control; nanoparticle selenium at 1 µg/mL (N1) and 2 µg/mL (N2); sodium selenite at 1 µg/mL (S1) and 10 µg/mL (S2). VCL: Curvilinear Line Velocity, VAP: Average Path Velocity, VSL: Straight Line Velocity, STR: Straightness, LIN: Linearity, ALH: Amplitude Of The Lateral Head, M: Mean, SD: Standard deviation, MED: Median, IQR: Interquartile range, Min: Minimum, Max: Maximum, ^AOV^: One-way ANOVA, ^WAOV^: Welch’s ANOVA, ^KW^: Kruskal–Wallis test. Different superscripts within the same column indicate significant differences (*p* < 0.05).

**Table 3:** Comparative evaluation of spermatological parameters of ram sperm samples in the study groups (*n* = 15 per group).

Variable	Groups	M	SD	MED	IQR	Min	Max	Statistic	*p*-Value
HOST Positive (%)	Control ^b^	32.60	9.17	35	11.5	14	46	F = 2.742	*p* = 0.035 ^AOV^
	N1 ^a^	50.87	18.91	55	27	24	85		
	N2 ^a^	44.27	16.71	40	19	22	78		
	S1 ^a^	48.00	17.82	45	20.5	21	82		
	S2 ^a^	42.27	17.25	39	18	17	77		
Viability (%)	Control ^b^	33.47	17.92	26	22.5	12	69	F = 4.121	*p* = 0.005 ^AOV^
	N1 ^a^	57.27	19.30	54	20.5	28	95		
	N2 ^a^	44.67	17.21	45	20.5	19	87		
	S1 ^a^	50.47	17.48	50	10	18	92		
	S2 ^b^	40.60	14.73	39	15.5	21	75		
Chromatin Condensation (%)	Control ^a^	15.53	1.77	15	2	12	19	F = 37.939	*p* < 0.001 ^AOV^
	N1 ^c^	5.80	2.34	6	2	2	12		
	N2 ^b^	9.60	3.04	9	3.5	4	15		
	S1 ^b^	8.87	2.42	9	2	5	14		
	S2 ^a^	13.80	2.60	14	2.5	9	18		
Head Abnormalities (%)	Control ^a^	9.47	3.27	9	3.5	4	16	F = 13.266	*p* < 0.001 ^AOV^
	N1 ^b^	3.13	1.19	3	2	1	5		
	N2 ^b^	5.07	2.49	5	3.5	1	9		
	S1 ^b^	4.80	2.48	4	4	1	10		
	S2 ^a^	7.00	2.95	7	3.5	1	12		
Midpiece Abnormalities (%)	Control	4.33	2.09	4	3	1	8	χ^2^ = 7.136	*p* = 0.129 ^KW^
	N1	2.80	1.66	2	2.5	1	6		
	N2	4.33	2.23	4	4.5	2	8		
	S1	4.20	2.65	3	4.5	1	9		
	S2	4.67	2.16	4	3	1	9		
Tail Abnormalities (%)	Control ^a^	11.27	3.56	11	2	6	18	F = 8.549	*p* < 0.001 ^AOV^
	N1 ^b^	5.87	2.36	6	1.5	1	11		
	N2 ^a^	8.73	3.77	10	5.5	3	15		
	S1 ^b^	6.80	2.60	7	2.5	2	10		
	S2 ^a^	9.87	1.85	10	2	6	13		
Normal Morphology (%)	Control ^c^	74.27	7.17	72	11	64	86	F = 13.393	*p* < 0.001 ^AOV^
	N1 ^a^	88.27	4.10	88	4.5	80	96		
	N2 ^b^	81.87	5.69	80	8.5	75	92		
	S1 ^a^	83.87	5.88	85	7	72	95		
	S2 ^b^	78.40	4.85	78	6	70	86		

Control; nanoparticle selenium at 1 µg/mL (N1) and 2 µg/mL (N2); sodium selenite at 1 µg/mL (S1) and 10 µg/mL (S2). M: Mean, SD: Standard deviation, MED: Median, IQR: Interquartile range, Min: Minimum, Max: Maximum, ^AOV^: One-way ANOVA, ^KW^: Kruskal–Wallis test. Different superscripts within the same column indicate significant differences (*p* < 0.05).

**Table 4:** Relative expression levels of the PRDX5 gene and TOS level in the fresh and frozen ram semen samples (*n* = 15 per group).

Variable	Groups	M	SD	MED	IQR	Min	Max	Statistic	*p*-Value
PRDX5 Expression	Control	2.40	2.88	1.15	1.69	0.37	10.63	χ^2^ = 1.639	*p* = 0.802 ^KW^
	N1	3.57	6.51	1.40	2.58	0.01	25.11		
	N2	2.90	3.85	2.02	2.74	0.10	15.45		
	S1	2.61	3.99	1.26	2.26	0.03	13.22		
	S2	5.28	11.01	0.23	4.79	0.02	42.37		
TOS Levels	Control	13.52	15.95	5.27	12.31	1.03	51.47	χ^2^ = 4.595	*p* = 0.331 ^KW^
	N1	6.54	6.67	4.48	5.17	0.83	23.05		
	N2	11.79	11.39	8.22	11.92	1.23	36.05		
	S1	10.97	9.35	8.96	10.37	0.73	29.50		
	S2	13.23	11.19	11.03	11.50	0.34	43.59		

Control; nanoparticle selenium at 1 µg/mL (N1) and 2 µg/mL (N2); sodium selenite at 1 µg/mL (S1) and 10 µg/mL (S2). M: Mean, SD: Standard deviation, MED: Median, IQR: Interquartile range, Min: Minimum, Max: Maximum, ^KW^: Kruskal–Wallis test.

## Data Availability

The data presented in this study are available on request from the corresponding author. The data are not publicly available as the original contributions presented in the study are included in the article. Further inquiries can be directed to the corresponding author.
